# Physical activity interventions in workplace health promotion: objectives, related outcomes, and consideration of the setting—a scoping review of reviews

**DOI:** 10.3389/fpubh.2024.1353119

**Published:** 2024-02-09

**Authors:** Andrea Schaller, Gerrit Stassen, Lukas Baulig, Martin Lange

**Affiliations:** ^1^Department of Human Sciences, Institute of Sport Science, University of the Bundeswehr Munich, Neubiberg, Germany; ^2^Institute of Movement Therapy and Movement-Oriented Prevention and Rehabilitation, German Sport University Cologne, Cologne, Germany; ^3^University of Police and Public Administration of North Rhine-Westphalia, Cologne, Germany; ^4^Department of Fitness and Health, IST University of Applied Sciences, Düsseldorf, Germany

**Keywords:** workplace health promotion, systematic review, physical activity, behavioral approach, environmental approach

## Abstract

**Background:**

The workplace is a recognized setting for promoting health among adults, and physical activity (PA) interventions are an integral part of workplace health promotion (WHP).

**Objectives:**

The present review of reviews aims to provide an overview of the main objectives and related outcomes addressed in WHP-related PA interventions, as well as the setting-specific aspects considered in the research field.

**Methods:**

A scoping review of reviews was conducted. Reviews were included if they were peer-reviewed, written in English, and focused on PA interventions conducted in the context of WHP. A literature search was conducted in PubMed, SPORTDiscus, and Web of Science. Reviews were included if they had been published after the year 2000. Information on the following dimensions was extracted: author, region, number of primary studies included, target group(s), PA interventions included, main objective(s), related outcomes, and setting-specific aspects.

**Results:**

A total of 17 reviews were included. Six reviews aimed at solely identifying the effectiveness of promoting daily PA and reducing sedentary behavior. Eleven reviews showed a combined approach considering physical activity behavior and/or health and job-related outcomes. Outcomes in the primary studies were heterogeneous. None of the reviews had an explicit definition of WHP and setting-specific information was very general and sparse. The reported setting-specific information was referred to the general importance of the workplace setting, the specific importance as an access route to target groups, and implementation aspects. Regarding the additional characteristics of the reviews, the selection of primary studies was restricted to a specific region in 2 of the 17 reviews in advance. Three reviews restricted the target group (sedentary workers, women, desk-based workers), while eleven reviews included working adults in general and, three reviews gave no information about the target group. Eleven intervention approaches of the reviews were behaviorally oriented, two focused solely on environmental interventions, and four reviews can be attributed to a combined approach considering behavioral and environmental interventions.

**Conclusion:**

For sustainable future developments, the present results indicate a strong need for conceptual consolidation of WHP in the research field of PA interventions. Therefore, both WHP and health-related PA interventions need to take a comprehensive approach comprising behavioral and environmental interventions.

## 1 Introduction

The workplace is a recognized setting to promote health among adults (1, 2). There is an opportunity to reach a heterogeneous target group for health promotion, especially health-related risk groups (3, 4) and socially deprived employees (3, 4). Furthermore, employees spend a long period of their day or life at work (1, 5). The current challenges in shortages of skilled workers, the increase in work density, and economic challenges are increasingly reinforcing the need to engage in employee health.

Workplace health promotion (WHP) programs can include organizational approaches, e.g., corporate policies, the development of networks (6), or environmental changes (e.g., staircase design), as well as behavior-related approaches, e.g., advice or coaching on lifestyle aspects (3). According to the Luxembourg Declaration, WHP comprises three key pillars: improving the work organization and the working environment, promoting active participation of the employees, and encouraging personal development (7). In a systematic WHP approach, behavioral measures (individual level) and environmental measures (organizational level) should be combined (7, 8). While corresponding multicomponent approaches are considered promising in WHP (9), isolated behavioral approaches are unlikely to be successful (10). The conceptual approach of WHP reflects the current state of research on the relevance of behavioral aspects and the respective mesosociological living environment – in this case, the workplace – as factors that influence health (11, 12). Thus, it can be derived from the Luxembourg Declaration (7) that WHP is much more than the “access route” to a target group (13, 14). Beyond, from a healthcare perspective, WHP pursues both risk reduction in the sense of prevention and competence development in the sense of health promotion (15).

Within the field of WHP, physical activity (PA) is a main field of action. Thereby, the concept and the related core objectives of PA promotion are multidimensional and comprise physical, mental and social health objectives, behavioral aspects, as well as living and working conditions (16, 17). In Germany, more than half of the environmental and around 70% of the behavior-related WHP measures are to be allocated to PA (18). The high proportion of PA interventions in WHP is due to the various positive effects of PA on health, which refer not only to physical capacity (19), but also to the prevention on the course of chronic diseases (19–23). Beyond, positive effects of PA on mental well-being (24, 25), social integration (26), social competence (27) and, regarding the work context, on promoting productivity (28), increasing workability (28), and reduction of sick leave (29, 30) are proven.

Taking into account the relevance of PA in WHP, the present review of reviews aims to provide an overview of the objectives of PA interventions, the main outcomes addressed, and how setting-specific aspects of the workplace are considered in the research field. The related research questions are:

1. What are the main objectives and outcomes addressed in workplace-related PA interventions?
2. How is the workplace setting considered in workplace-related PA interventions?

## 2 Methods

A scoping review of reviews (SRR) was conducted to take a superordinate perspective by taking reviews into account while reducing the amount of literature. An SRR approach can elucidate current research directions and conceptual ambiguities, providing an overview of research activities and possible gaps on a particular topic, and addressing exploratory research questions (31–34). This SRR was conducted following the Preferred Reporting Items for Systematic Reviews and Meta-Analyses Extension for Scoping Reviews (PRISMA-ScR) (35) and was registered prospectively on the Open Science Framework on June 8, 2022.^1^

### 2.1 Eligibility criteria

Reviews were included if they 1) were peer-reviewed, 2) were written in English, 3) focused on PA interventions, and 4) were conducted in the context of WHP. Appropriate article types were systematic review, meta-analysis, meta-synthesis, scoping review, rapid review, and narrative review. The eligibility criterion of a WHP context was assessed based on the target population of working adults and/or terms such as “health” and “health promotion” in combination with “workplace-related,” “in/at the workplace” and other similar terms in the title, abstract, and description in the background, review objective, or methods.

Exclusion criteria were 1) interventions only assessing PA as an outcome without a PA intervention component, 2) reviews with mixed focuses that examined combined approaches or dealt with PA promotion as only one aspect or intervention component, respectively (e.g., multidimensional approaches additional consideration of, among others, sleep or nutrition or stress or substance use interventions), 3) reviews including also formative, mixed-method or qualitative studies and not only interventions studies, and 4) reviews on PA interventions on patient populations or the treatment of specific diseases. We limited the search period to published studies between January 2000 and November 2023.

### 2.2 Information sources and search

A computerized systematic literature search was conducted in PubMed, SPORTDiscus, and Web of Science and finalized in November 2023. Search terms related to physical activity, the workplace setting, health promotion, and intervention research were used with operators (“OR,” “AND,” and “NOT“) and truncations (“*”) with appropriate adjustments for each database. Articles were imported into the literature management program Rayyan (36). After removing all duplicates, two reviewers (GS and LB) independently screened all titles/abstracts in the first step and full texts in the second step based on the eligibility criteria. Any disagreements were resolved by consensus or consultation with a third reviewer (AS).

### 2.3 Data charting and synthesis of results

**Figure 1:**
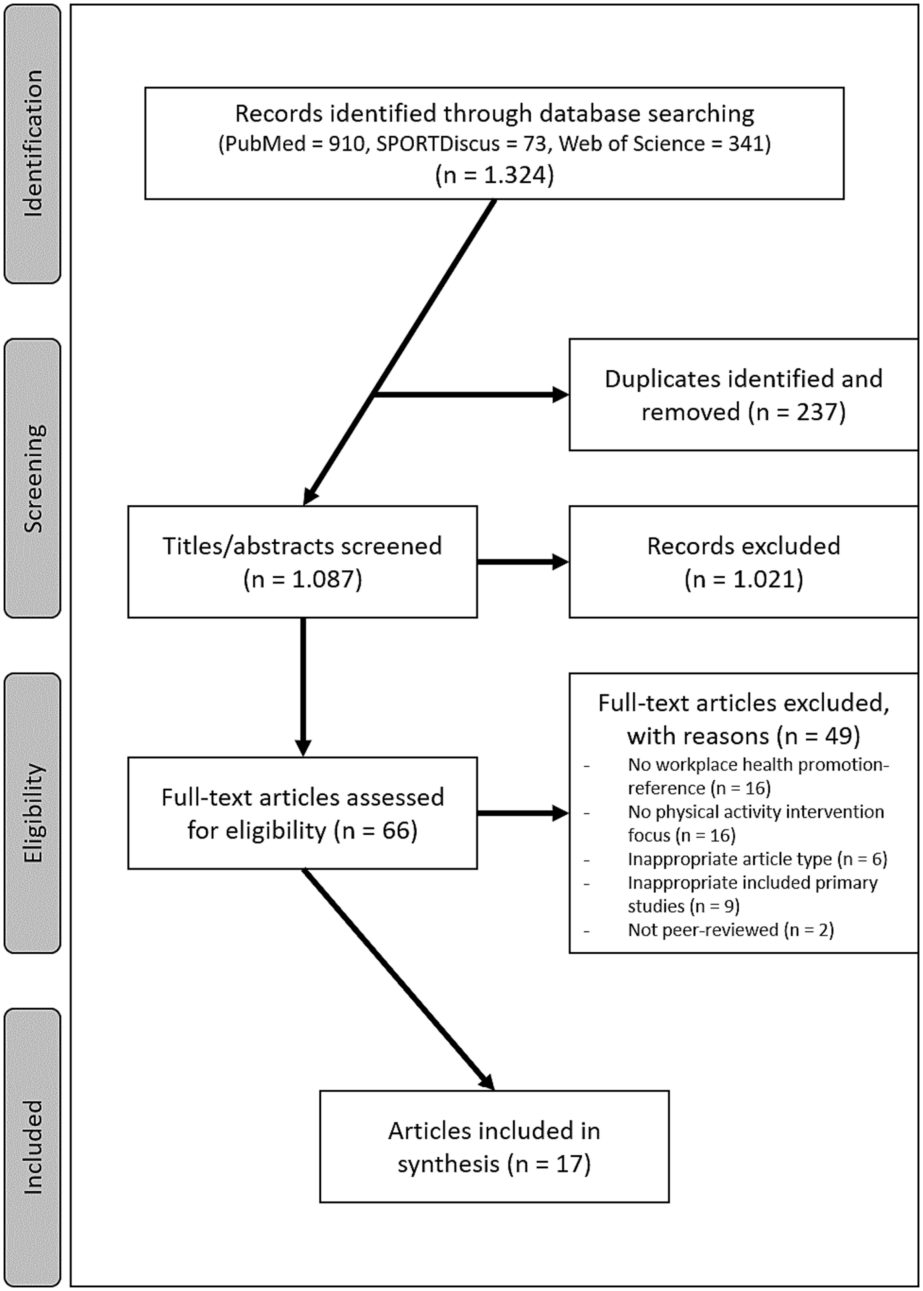
Flow diagram illustrating the search and selection process.

Two reviewers (AS and GS) read all included full texts and extracted the following information from the reviews: first author *(name; year)*, region *(region defined in the review/regions of the primary studies),* number of included primary studies in the review *(n),* target group *(of the review/participants in the primary studies),* PA interventions *(interventions included in the review (behavioral; environmental)/intervention approaches of the primary studies),* main objective(s) of the review, related outcomes in the primary studies *(physical activity-related/health-related/job-related),* information on the consideration of the workplace setting *(implementation aspects/importance as an access route to employees/general significance of the setting).* The information extracted was categorized within the results table and summarized narratively (see Figure 1, Table 1).

**Table 1:** Characteristics, main objectives, and information on the conceptual basis for workplace health promotion.

#	Author	Region defined in the review/regions of the primary studies	Number of included primary studies	Target group(s) of the review/branches of the primary studies	Physical activity intervention included in the review/intervention approaches of the primary studies	Main objective(s) of the review	Related outcomes in the primary studies	Information on the consideration of the workplace setting
1	Abdin et al. (37)	Not specified/Europe, Australia	5	Working adults/university settings, small- to medium-sized organizations	*Behavioral*: Face-to-face interventions/exercise, yoga, walking	Effectiveness of PA interventions for improving psychological well-being	*Health-related outcomes*: Stress, life purpose, life satisfaction, subjective wellbeing	No information
2	Bordado Sköld et al. (38)	Not specified / Europe, North America, Japan, Australia	22	Working adults/employees, health personnel in hospitals or nursing homes, laboratory technicians, and other manual job settings	*Behavioral*: Minimum weekly workplace exercise/cardio training and aerobics, strength and resistance training, yoga, walks	Effects on the psychosocial working environment and mental health among employees	*Health-related outcomes*: Mental health and/or psychosocial environment	*Implementation aspects*: Interventions at the workplace or during working hours
3	Buckingham et al. (39)	Not specified / United States, Australia, Canada, Netherlands, Belgium, Singapore, Finland, Norway	25	Working adults/academic and academic medical institutions, healthcare, health insurance, wellbeing improvement, property and infrastructure, pension Insurance, financial services, road maintenance, haulage	*Behavioral and/or environmental*: Mobile health technology, including wearable activity monitors and smartphone applications/standalone mobile health interventions, multi-component interventions; exclusive workplace or more comprehensive lifestyle interventions	Effectiveness, feasibility, and acceptability of mHealth interventions in the promotion of physical activity and reduction of sedentary behavior in the workplace	*Physical activity-related outcomes*: Sedentary time, daily steps, weekly physical activity, weekly exercise, intensity-specific physical activity	*Importance as an access route to employees*: Recruitment at the workplace and/or the intervention was delivered in the workplace *Implementation aspects*: Exclusive workplace or a wider lifestyle intervention
4	Burn et al. (40)	Not specified / Denmark, United Kingdom, Sweden, Norway, Netherlands, Turkey, Canada, United States	12	Working adults/office workers, pharmaceutical company workers, construction workers, care workers, cleaners, poultry processing workers	*Behavioral*: Interventions of at least moderate intensity / Aerobic (walking: nature walk; stair climbing; running), Resistance (kettle bells, lower body strengthening, theraband exercises), multi-component training (cycling, rowing, and resistance training)	Effects on cardiorespiratory fitness (CRF)	*Health-related outcomes*: Peak oxygen consumption (*V*O2peak), CRF (as measured by actual or predicted *V*O2peak)	*Implementation aspects*: The workplace as a relatively controlled setting Prescription and delivery in the workplace or commenced from the workplace
5	Chau (41)	Not specified / not reported	6	Working adults/universities, mid- to large-sized workplaces, occupational health care units	*Behavioral*: Interventions to increase energy expenditure: increase physical activity or decrease sitting/tailored physical activity advise or counseling, physical activity counseling plus fitness testing, weekly email messages, pedometers	Effectiveness of workplace interventions to reduce sitting	*Physical activity-related outcomes*: Specific measure of sitting or activities ≤1.5 METs (self-report or objective; including measures of sitting with or without duration)	*Implementation aspects*: The interventions were carried out in a workplace setting
6	Commissaris (42)	Not specified / not reported	40	Not reported / not reported	*Behavioral and/or environmental*: Interventions during productive work / alternative workstations, interventions promoting stair use, personalized behavioral interventions	Effectiveness in reducing sedentary behavior and/or increasing physical activity	*Job-related outcomes*: Work performance *Physical activity-related outcomes*: Metabolic and physiological responses	*Implementation aspects*: The interventions were implemented during productive work in a workplace
7	Forberger et al. (43)	Not specified / United Kingdom, Belgium, France, Spain, Switzerland, United States, Australia, Canada, Japan, Singapore	26	Working adults/ universities, public authorities, health insurance companies, private companies (brewing company, hospital, IT company, telecommunication, water supply)	*Environmental*: Interventions using nudges/software package for Sitting Pad, information, and guidelines, WatchMinder that vibrates every 30 min, poster prompts, office clock with reminder function, meetings on foot	Analyze WHP interventions aiming at increasing PA or reducing SB using nudges	*Physical activity-related outcomes*: Increase PA. decrease SB	*Importance as an access route to employees*: Facility-based approaches such as WHP programs as a promising strategy to increase daily PA in the adult population *General importance of the setting*: Life course perspective of WHP, targeting a significant portion of the population throughout their working lives
8	Freak-Poli et al. (44)	Various high-income countries	14	Working adults / diverse workplaces (from offices to physical workplaces)	*Behavioral*: Multi-component health promotion interventions with a pedometer component for the entire duration of the intervention	Effectiveness to increase physical activity and improve long-term health outcomes	*Physical activity-related outcomes*: Metabolic equivalents (METs), step count, METs for moderate and vigorous activity combined, incidental activity (incorporated into work or leisure time), sedentary behavior *Health-related outcomes*: Cardiovascular disease and type 2 diabetes risk factors, Anthropometric measures, blood pressure, hypertension, resting heart rate, biochemical measures, blood cholesterol, disease risk scores, type 2 diabetes risk, quality of life, social support, satisfaction with life, adverse effect	*General importance of the setting*: The workplace was described as a key setting for health promotion and disease prevention Opportunities for employers to improve worker health, reduce absenteeism, and increase productivity and benefit for the employee *Importance as an access route to employees*: Opportunity to access groups of participants in their daily lives
9	Hutcheson et al. (45)	Not specified / Australia, North America, Europe	15	Working adults/call center, physical activity research center, health promotion office, university employees, health agency employees, government agency employees	*Environmental*: Worksite interventions targeting sedentary behavior	Identify work site–based, environmental interventions to reduce sedentary behavior	*Physical activity-related outcomes*: Sedentary behavior: inclinometers, accelerometers, self-report questionnaires	*General importance of the setting*: The work site setting as an important setting for health promotion
10	Lusa et al. (46)	Not specified / United States, Netherlands, Finland, Denmark, Spain, Canada, Australia, United Kingdom, Turkey, Germany, Slovenia, Japan, Saudi Arabia	29	Sedentary workers / not reported	*Behavioral*: Interventions aiming at decreasing sedentary time at work or increasing the amount of PA at work or during leisure time / physical exercise-oriented interventions, physical exercise and/or breaks, physical exercise and/or workstation activities	Promoting work ability by increasing physical activity at workplaces	*Job-related outcomes*: Work productivity, sickness absence, work ability, work performance	*Implementation aspects*: The interventions were carried out in a workplace setting or organized by the employer Interventions were performed a) at the workplace, b) both at workplaces and during leisure time, c) in a laboratory or a simulated situation
11	Moreira-Silva et al. (47)	Not specified / not reported	12	Not reported / not reported	*Behavioral*: Interventions in the workplace	Effectiveness in reducing musculoskeletal pain	*Health-related outcomes*: Back pain, CRF, aerobic capacity, well-being, muscle pain, back pain, complaints from neck and shoulders, headache, extension and flexion strength of the upper extremities, subjective health complaints *Job-related outcomes*: Job satisfaction, sick leave	*General importance of the setting*: To promote health by reducing health risks and actively preventing disease incidence
12	Nestler et al. (48)	Not specified / not reported	15	women aged 18 to 65 years / not reported	*Behavioral*: Training/gym machine training, exercise bands, body perception training, body weight training, static arm-holding exercise, endurance running	Effects on muscle strength, physical performance ability, and health-related parameters	*Health-related outcomes*: Body composition, musculoskeletal pain, subjective well-being	*General importance of the setting*: High potential for health-promoting activities
13	Pereira et al. (49)	Not specified/ not reported	9	Working adults / not reported	*Behavioral and/or environmental*: Any onsite workplace structured health-enhancing physical activity (HEPA) programs during or outside of paid work time but distinct from work-related PA / strengthening exercises, aerobic exercises, physical training, walking routes, walking while working, integrated health programs including educational sessions, therapeutic yoga	Effects from the employer’s perspective	*Job-related outcomes*: productivity measurement *Job-related outcomes*: Quantitative measurements of job performance, workability	*General importance of the setting*: WHP as an important component of an organization’s business plan to improve worker health and productivity *Implementation aspects*: On-site workplace programs outside of regular work duties
14	Taylor et al. (50)	Not specified / not reported	13	Desk-based workers / not reported	*Behavioral and/or environmental*: Prompt software programs installed as behavioral change interventions/software, reminder systems, computer prompt	Reducing sedentary behavior and promote physical activity	*Physical activity-related outcomes*: Sedentary behavior and/or physical activity at work	*General importance of the setting*: Optimal venue for health promotion interventions (established structures, reaching the target group for extended periods, mobilization of multiple tools and resources, established communication channels, modification of environments) *Implementation aspects*: Interventions during work hours and delivered through a work personal computer or laptop
15	Vuillemin et al. (51)	Europe / United Kingdom, Finland, Belgium, Norway, Spain, Switzerland, Netherlands, Sweden, Denmark, Germany	33	Working adults / not reported	*Behavioral*: Interventions increasing physical activity of employees in a workplace/counseling, exercise training (aerobic fitness and muscular training), active commuting, walking interventions, stair use, multi-component interventions	Effectiveness of physical activity promotion interventions in the worksite	*Physical activity-related outcomes*: Habitual physical activity level *Health-related outcomes*: Physical fitness (CRF, strength); obesity-related outcomes (BMI, body weight, percentage body fat, waist circumference, waist-to-hip ratio)	*General importance of the setting*: Important setting to implement programs and strategies to promote physical activity and prevent body weight gain and obesity *Implementation aspects*: Interventions in a worksite setting (including commuting) The worksite as a relatively controlled environment where a substantial proportion of the adult population can be reached
16	Amatori et al. (52)	Not specified / not reported	7	Working adults / employees in universities, hospitals, or office settings	*Behavioral*: High-intensity training (HIT) programs within the workplace and tested at least one physiological, psychological, or work-related outcome (training type; modalities; training frequency; session duration; exercise Intensity; intervention duration)	Summarize the evidence about the feasibility and effectiveness of HIT interventions in the workplace setting for improving health- and work-related outcomes	*Physical activity-related outcomes*: Body composition, CRF, muscle strength, blood pressure, haematochemical parameters *Health-related outcomes*: Mental wellbeing, health-related quality of life, stress, anxiety, motivation and self-efficacy to exercise *Job-related outcomes*: Work productivity, job satisfaction	*General importance of the setting*: Potential solution to counteract the adverse effects of prolonged sitting time, sedentary behavior, and monotonous and/or strenuous physical tasks Exercise interventions in the workplace represent a viable approach to increasing employees’ health *Importance as an access route to employees*: Lack of time, work schedule conflicts, low perceived self-efficacy, and lack of motivation were reported to be the most important barriers to workplace exercise participation Strategies to overcome these barriers: opportunities to exercise throughout the workday and organizing frequent group exercise classes *Implementation aspects*: exercise programs are generally not integrated into a work environment Rather constrained to a “would be nice to have” add-on Many programs fail, mainly due to poor integration in the work environment, perceived lack of time, low self-efficacy, and lack of motivation
17	Larinier et al. (53)	Not specified*/*United States, Sweden	3	Healthy adult employees /military recruits; construction or manual workers	*Behavioral*: Warm-up defined as short bout of exercise realized before work and aiming to improve muscle dynamics to prevent injury and to prepare the worker to learn its task / passive stretching intervention targeting the whole body; a combination of exercises based on dynamic movements, dynamic flexibility, strengthening, agility, and plyometric exercises	Assess the effects of warm-up interventions implemented in the workplace on work-related musculoskeletal (WMSDs), physical and psychosocial functions	*Health-related outcomes*: Pain, discomfort, fatigue, quality of life, psychosocial stress at work, injury rate *Physical activity-related outcomes*: Physical functions, leisure time activity *Job-related outcomes*: Job satisfaction, motivation at work, xperienced workload	*General importance of the setting*: Great potential for improving health and preventing WMSDs *Importance as an access route to employees*: Perfect area to reach and to raise awareness of a large number of workers

## 3 Results

The systematic search resulted in 1.324 reviews. After removing duplicates, screening titles, abstracts, and full texts, 17 reviews met the inclusion criteria (see Figure 1).

### 3.1 Main objectives of the reviews

Six reviews pursued the objective of identifying solely the effectiveness of PA interventions on promoting PA and/or reducing sedentary behavior (39, 41–43, 45, 54). Two reviews focused on the effects of PA interventions on improving psychological well-being and mental health (37, 38, 50, 52) and two assessed workability (46, 53). One review each showed a fitness approach (40, 49) or focused musculoskeletal pain (48). Five reviews showed a combined approach regarding the objectives, considering physical activity behavior and health outcomes (38, 40, 44, 47, 52). The outcomes of the respective primary studies could be assigned to the following categories: physical activity-related outcomes (38, 39, 41–45, 47, 52, 54), health-related outcomes (37, 38, 40, 44, 47–50, 52), and job-related outcomes (38, 41, 46, 48, 52, 53).

### 3.2 Consideration of the workplace setting

Although relevant information was drawn from all sections of the reviews (background, methods, discussion) regarding the consideration of the workplace, very little information was found. The available information from the reviews was classified into three categories: general consideration of the setting, the workplace as an access route to employees, and implementation aspects. Ten reviews noted the general importance of the workplace as a critical setting for health promotion and disease prevention (38, 40, 42–47, 52). The relevance was mainly explained by health effects for employees, with two reviews also arguing from a company-related perspective (46, 47). Implementation aspects were considered in ten reviews (37–39, 41, 44–46, 49, 53, 54). Three times it was specified that the interventions were carried out solely during working hours (37, 41, 45) and eight times that they should be carried out at the workplace (37, 39, 41, 45, 46, 49, 53, 54). Regarding implementation, two reviews described the workplace as a relatively controlled setting for implementing a PA intervention (44, 49) and three reviews focused on interventions conducted at workplaces and during leisure time (46, 53, 54). The importance of WHP as an access route to a target group in their daily lives was explicitly mentioned in five reviews (38, 42, 47, 52, 54).

### 3.3 Further characteristics of the reviews

Two of the 17 reviews restricted their selection of primary studies in advance to a specific region [various high-income countries (47), Europe (44)]. In eight reviews, there was no regional restriction in the search strategy, but the countries where the primary studies were conducted were reported in the results section (37, 42, 43, 49, 50, 52–54). Seven reviews (38–41, 45, 46, 48) did not report the region of the studies or the study selection at any point. The number of studies included ranged from 3 (50, 52) to 40 (41). In three reviews, a search restriction was made to a specific target group (sedentary workers, women, desk-based workers) (40, 45, 53). Ten reviews included working adults in general but reported the branches or sectors of the primary studies included (37–39, 42, 43, 47, 50, 52, 54). Two reviews gave no information about the target group (43, 48). Eleven intervention approaches of the reviews were behaviorally oriented (37, 39, 40, 44, 47–50, 53), two reviews were focused solely on the environment (42, 43), and four reviews can be attributed to a combined approach considering behavioral and environmental intervention approaches (41, 45, 46, 54).

## 4 Discussion

This review of reviews provides an overview of the objectives, related outcomes, and how settings-specific aspects in workplace-related PA interventions were considered. Overall, information on the consideration of the setting in the reviews was sparse. The information reported was somewhat related to general aspects regarding the importance of the WHP setting and/or an access route to the respective target group and implementation aspects. There was substantial heterogeneity in terms of the objectives of the reviews and the resulting outcome variables included in the primary studies. Regarding additional characteristics, the region and the target group were rarely specified or restricted, and most reviews referred to behavioral PA interventions.

It is widely known that WHP and PA interventions should go beyond addressing purely functional or biomedical outcomes on the individual level. In the field of health promotion, there is a common understanding that the concept of health refers not only to the physical dimension but also to the psychological and social dimensions. Though health is more than the mere absence of complaints, disorders, or diseases (51, 55), it is considered a resource for daily life (2). Based on the Ottawa Charter, interventions in health promotion address “the process of enabling people to increase control over, and to improve, their health” (2). The objectives addressed in the reviews generally indicate that some multidimensionality of PA interventions is apparent in the research field of WHP. Even though most reviews focus on the effects of PA interventions on PA promotion or the reduction of sedentary behavior, also effects on mental health or workability were examined. This is underlined by the outcomes assessed in the included primary studies (PA-related, health-related, and job-related outcomes) (38, 41, 46, 48, 53). Nevertheless, less than half of the reviews pursued a combined and multidimensional approach by considering PA behavior and health outcomes. Beyond this, no systematic consideration of the biopsychosocial approach is apparent in the reviews. This finding is consistent with two reviews on WHP in Nordic countries, which elaborated that the research field tends to focus on a biomedical perspective, that the workplace setting is primarily seen as an access to the target groups, and that the majority of the studies focused on pathogenic outcomes and individual risk factors, rather than environmental changes (28, 56). The present review of reviews also showed that PA interventions in WHP are primarily behavior-oriented. This may indicate that PA interventions do not meet the requirements of an integrated WHP, which combines organizational and behavioral approaches (7, 8). Besides, this does not comply with the recommendations for PA promotion suggesting conducting multicomponent PA interventions combining environmental measures on the structural and process levels (e.g., creating an infrastructure to promote PA; the possibility of participation during working hours) and behavioral measures (e.g., courses and exercise programs) (57).

As WHP can be considered a complex intervention in a complex setting (58), it was astonishing that, based on our findings, the reviews were not based on an explicit definition of WHP. Even if there is no international definition of WHP at present, it would be beneficial for the respective reviews to define what is meant by WHP or how it was defined in the search strategy. Beyond this, no detailed information on how the workplace setting was considered was elaborated in the reviews. The sparse information in the included reviews on the consideration of setting-specific aspects in PA interventions in WHP indicates that the term WHP might instead be used as an umbrella term and not as a theoretical foundation or defined framework condition of the PA interventions. From a meso-sociological perspective, WHP-related organizational framework conditions of the respective PA intervention should be considered (59). Therefore, PA interventions at the workplace should not only be seen as a training intervention at a specific location (the workplace), but it is necessary to systematically implement them into the respective organization and establish new structures. This includes, amongst others, necessary personnel and financing aspects and implementing the PA intervention in the operational process. Against the same background, it is surprising that the respective employee target group or the corporate branch was not specified in more detail. Both are key components in developing and evaluating complex interventions (60–62). The standard application of implementation frameworks could substantially contribute here (63). For example, a hybrid approach that combines elements of clinical effectiveness and implementation research might be promising (64). Curran et al. (64) distinguish between three types of hybrid models, which, transferred to the field of workplace-related PA interventions, could be characterized as follows: (1) testing the effects of a PA intervention on relevant outcomes on the individual level, while gathering information on implementation, (2) dual testing of the effects of the PA intervention on the individual level and implementation strategies, and (3) evaluating an implementation strategy while gathering information on the effect of the PA-intervention on relevant outcomes on the individual level. However, of the 17 reviews included in our review of reviews, only one showed a hybrid approach (38).

From a macro-sociological perspective, the research field should also consider the respective national welfare law framework and related quality criteria regarding WHP [e.g., (65)]. Against this background, it is surprising that most reviews have included studies from different countries and even continents alike. As there are various national legal bases for WHP, this underlines the great heterogeneity and inconsistent use of WHP as an umbrella term. Different national welfare law framework for WHP might affect, amongst other things, the intervention design, but also the goals of a PA intervention. Even within Europe, occupational health and safety policies differ considerably. Some member states require no action on promoting health at work, while others show fragmented attention to WHP in their national policy (66). In contrast, six countries (Austria, Denmark, Finland, Germany, Sweden, and the United Kingdom) provide clear national guidelines specific to WHP. Thus, Germany has even adopted national regulation for WHP with a legal obligation for the statutory health insurers to finance WHP to a certain extent if they comply with defined quality criteria (66).

Although the present review of reviews makes an important contribution to the future development of conceptual approaches in WHP-related PA promotion, it is important to point out some limitations. First, the analyzes were based on the researchers’ prior understanding of the topic under investigation, especially health-related PA and WHP. In this respect, the analysis does not aim to verify or objectify but to contribute to research on PA interventions in WHP. Second, to answer the research question on the consideration of the setting in the reviews, we took a comprehensive approach to data extraction and considered all relevant information from the respective review, regardless of whether it was in the background, methodology, results, or discussion. This can be considered both a strength and a limitation. On the one hand, this weakens the systematic nature of the search; on the other hand, against the background of the current approach in this subject area, this offers a first opportunity to elaborate on the sparse setting-specific information. Third, the inclusion criteria were limited exclusively to reviews with PA interventions. Multidimensional intervention approaches (e.g., PA interventions in combination with nutrition and/or stress management services) were not included. Finally, the scoping review of reviews approach does not allow conclusions to be drawn about the description of the conceptual foundation and setting-specific information regarding WHP in the primary studies.

## 5 Conclusion

How the setting-specific aspects are taken into account in WHP-related PA interventions fundamentally influences the future research. This review of reviews underlines a strong need for conceptual consolidation of WHP and the consideration of the setting in the research field of PA interventions. Thereby, both WHP and health-related PA-interventions take a comprehensive and integrated approach considering behavioral and environmental interventions. The development of evidence in WHP-related PA interventions is considerably more difficult as long as there is a lack of theory-based PA interventions in WHP. Furthermore, it is of utmost importance to obtain and consider precise information on the meso- and macro-sociological context factors to sustainably implement and/or transfer PA intervention in WHP. For this reason, future studies should also explicitly report on the respective national legal bases for WHP.

## Data availability statement

The original contributions presented in the study are included in the article/supplementary material, further inquiries can be directed to the corresponding author.

## Author contributions

AS: Conceptualization, Data curation, Formal analysis, Funding acquisition, Methodology, Project administration, Resources, Visualization, Writing – original draft, Writing – review & editing. GS: Conceptualization, Data curation, Formal analysis, Methodology, Writing – review & editing. LB: Data curation, Formal analysis, Writing – review & editing. ML: Writing – review & editing.

## Footnotes

^1^ https://osf.io/w8rq5
